# The value of pre-operative outpatient flexible sigmoidoscopy in patients with deep infiltrating endometriosis

**DOI:** 10.52054/FVVO.15.2.076

**Published:** 2023-06-30

**Authors:** T Sewell, M Orchard, O O’Donovan, R.J. Longman

**Affiliations:** University Hospitals Bristol and Weston NHS Foundation Trust, Trust Headquarters, Marlborough Street, Bristol, BS1 3NU, UK

**Keywords:** Sigmoidoscopy, deep infiltrating, endometriosis

## Abstract

**Background:**

Deep infiltrating endometriosis (DE) is a particularly severe disease which affects 10-20% of women with endometriosis. 90% of DE is rectovaginal and when suspected, some clinicians have suggested the routine use of flexible sigmoidoscopy to identify intraluminal disease. We aimed to assess the value of sigmoidoscopy prior to surgery for rectovaginal DE, both in terms of diagnosis and planning management.

**Objectives:**

We aimed to assess the value of sigmoidoscopy prior to surgery for rectovaginal DE.

**Material and Methods:**

A retrospective case series study was performed from a consecutive cohort of patients with DE referred for outpatient flexible sigmoidoscopy between January 2010 and January 2020. All patients were under the care of a specialist endometriosis multidisciplinary team.

**Main outcome measures:**

The primary outcome measure was the incidence of luminal disease.

**Results:**

102 consecutive cases were analysed with no cases confirming intraluminal disease. Non-specific evidence of endometriosis such as tight angulation of the bowel was found in 36.3%. Following sigmoidoscopy 100 patients proceeded to surgery and the risk of bowel resection during surgery was 4%.

**Conclusions:**

Due to the low incidence of luminal endometriosis, performing sigmoidoscopy routinely is of limited benefit. We recommend the selective use of sigmoidoscopy where serious pathology such as colorectal neoplasia is considered or to determine the location of endometriosis lesions which aids subsequent resectional surgery planning.

**What is new?:**

This large case series details a very low incidence of intraluminal disease and makes recommendations for the specific scenarios where flexible sigmoidoscopy should be used.

## Introduction

Endometriosis is defined as the presence of endometrial glands and stroma like lesions outside of the uterus (Giudice and Kao, 2004). It is a chronic idiopathic condition with an estimated prevalence of 10-15% in women of reproductive age (Giudice and Kao, 2004). Endometriosis encompasses three main clinical variants which often overlap; superficial endometriosis, ovarian endometriosis (endometrioma) and deep infiltrating endometriosis (DE) (Nisolle and Donnez, 1997). Definitions vary, but DE may be defined as lesions extending 5mm or more below the peritoneal surface (Cornillie et al., 1990) and is present in up to 20% of women with endometriosis (Nisolle and Donnez, 1997). Of those with DE, 90% have rectovaginal lesions affecting the tissue adjacent to, or occasionally directly involving the rectum and/or rectosigmoid colon (De Cicco et al., 2011). The remaining 10% of DE primarily affects other areas of the bowel as well as the ureters or bladder. DE often produces severe symptoms which may be challenging to manage. The most characteristic symptoms include dysmenorrhoea, dyspareunia, dyschezia, rectal bleeding, dysuria, non-cyclical pelvic pain, sexual dysfunction, and subfertility. In addition, the psychological impact from this chronic disease may be significant and should not be underestimated.

Diagnostic laparoscopy remains an important investigation for the diagnosis of endometriosis. However, it’s use is limited when exploring rectovaginal DE as the pouch of Douglas is often obliterated meaning further exploration would require extensive surgical dissection. Due to the invasive nature of diagnostic laparoscopy, imaging is nearly always recommended, and ultrasonography is often the initial imaging modality of choice (Rolla, 2019). Transvaginal ultrasound (TVUS) is particularly useful in diagnosing endometriomas, however, as with other imaging modalities, it will not reliably detect superficial disease. In experienced hands, TVUS can reliably detect rectovaginal DE (sensitivity and specificity 91% and 97% respectively (Guerriero et al., 2015), and MRI has similar accuracy (sensitivity and specificity of 85% and 96% respectively) (Guerriero et al., 2018).

When rectovaginal DE is suspected, routine flexible sigmoidoscopy has been suggested, with some authors reporting high diagnostic yield as well as high sensitivity and specificity for the detection of intraluminal endometriosis (Lukovich et al., 2017; Kim et al., 2011; Abrao et al., 2015). Lukovich et al. (2017) studied 383 sigmoidoscopies performed in patients with endometriosis and found intraluminal endometriosis in 4.9% of cases and “secondary signs” such as rigidity, bowel kinking and pain in 38-57% of cases. This additional information can be used to aid pre-operative patient counselling and surgical planning. However, it is a relatively invasive test which can be uncomfortable and carries a small risk of complications including bowel perforation. To date, relatively few studies have evaluated the value of flexible sigmoidoscopy in this setting. We aimed to assess the value of flexible sigmoidoscopy prior to surgery for rectovaginal DE.

## Methods

A retrospective observational case series study was performed from a consecutive cohort of patients referred for outpatient flexible sigmoidoscopy prior to planned surgery for rectovaginal DE. All patients were under the care of a specialist multidisciplinary team based at a single UK hospital providing a tertiary referral service for women with suspected or confirmed DE. Amongst others, the team included gynaecologists specialising in endometriosis, specialist nurses and a colorectal surgeon with a specialist interest in the management of endometriosis involving the bowel. Patients were referred to the team following a confirmed or suspected diagnosis of endometriosis based upon prior imaging or recent diagnostic laparoscopy where rectovaginal DE had been diagnosed but not excised. Additional investigations such as TVUS, MRI or further diagnostic laparoscopy were requested at the discretion of the team based upon individual clinical need. The decision to undergo surgery was made by the team in conjunction with the patient. It was the routine practice of the team to refer all patients planning surgical management of rectovaginal DE for flexible sigmoidoscopy. In all cases, the sigmoidoscopy was performed prior to their surgery date and was not specifically timed with menses.

Patients with rectovaginal DE undergoing surgery were identified from the multidisciplinary team’s endometriosis database. The study inclusion criteria were all patients of any age who underwent sigmoidoscopy prior to planned surgery for confirmed or suspected rectovaginal DE within a 10-year time period (January 2010 – January 2020). Patients opting for conservative or medical management were not routinely referred for flexible sigmoidoscopy and therefore excluded. Patients who declined to undergo sigmoidoscopy prior to their surgery were also excluded. Finally, patients with solely other forms of endometriosis (such as superficial disease, endometrioma or non-rectovaginal DE), non-endometriosis related pathology and those with only incomplete data available were excluded from the study.

Data collection involved review of the case notes and internal hospital electronic medical records for all procedures performed over the study period. Specifically, the procedure record from the sigmoidoscopy as well as the operation notes were reviewed alongside extensive review of all correspondence and clinic appointments between the patient and the medical team.

All patients in the cohort were referred for sigmoidoscopy for the purpose of excluding luminal endometriosis and to aid with patient counselling prior to surgery. All patients who were referred consented to undergo the procedure. In all cases per rectum digital examination was performed at the time of sigmoidoscopy.

Data were collected on patient’s age, body mass index (BMI), comorbidities, previous pelvic surgery, symptoms, imaging findings, flexible sigmoidoscopy outcomes, surgical findings and operation performed. The primary outcome measure was the incidence of luminal disease at flexible sigmoidoscopy. Secondary outcome measures consisted of other pathology found during flexible sigmoidoscopy, technical feasibility, and complications.

Data analysis was performed using standard techniques for normally distributed data comprising of the calculation of means, standard deviation and percentages using Microsoft Excel (Microsoft Corporation. (2018)). The project was registered and approved with the local Trust’s research department. Analysis was performed on retrospectively collected fully anonymised data and therefore, as per national and international guidelines, ethics committee approval was not required.

## Results

111 consecutive cases were identified from the endometriosis database as meeting the inclusion criteria. Of these 9 (8.1%) were excluded from the analysis due to incomplete data sets meaning 102 patients with suspected or confirmed rectovaginal DE were analysed. The patient characteristics are shown in Table I. All sigmoidoscopy procedures were performed primarily to diagnose or exclude evidence of luminal endometriosis.

**Table I:** Operative pelvic surgery includes all laparoscopic or open procedures involving the pelvis but excludes purely diagnostic laparoscopy.

Characteristic (N = 102)	Mean (Standard Deviation)
Age	33.9 (6.3)
BMI	26.8 (4.8)
	n (%)
Previous Operative Pelvic Surgery	59 (57.8%)
Previous Diagnostic or Operative Pelvic Surgery	94 (92.2%)

The patient symptoms at presentation to the specialist endometriosis service are shown in Table II. Rectovaginal DE was diagnosed by a combination of ultrasound, MRI, and diagnostic laparoscopy. 91 patients (89.8%) had ultrasound imaging and of those scans 31.9% showed evidence of endometriosis. This relatively low rate of diagnosis with ultrasound is likely due to a lack of specialist endometriosis ultrasound operators until the last 20 months of the study period when a new clinician joined the team. The most common ultrasound finding was an endometrioma (65.5%) followed by rectovaginal nodules (17.2%) and adherent non-mobile ovaries (17.2%). MRI scans were performed in 23 (22.5%) patients with 73.9% of scans showing evidence of endometriosis. In contrast to ultrasound imaging, the most common endometriosis-related finding on MRI were rectovaginal nodules (70.6%) with endometriomas found in the remaining 29.4%. Finally, 94 (92.2%) patients had undergone a diagnostic laparoscopy with the majority of these procedures performed in other units prior to referral to the specialist endometriosis team. Therefore, laparoscopy represented the principal method of diagnosis for rectovaginal DE in this study.

**Table II:** Subfertility was defined as no documented pregnancy after 1 year of unprotected intercourse or those currently having investigation and/or treatment for subfertility. In addition to the 23 patients with confirmed subfertility there were 31 patients (30.4%) who had not attempted to conceive.

Symptom (N =102)	n (%)
Non-cyclical pelvic pain	75 (73.5%)
Dysmenorrhoea	68 (66.7%)
Dyspareunia	65 (63.7%)
Rectal Bleeding	13 (12.7%)
Dyschezia	58 (56.9%)
Altered bowel habit (diarrhoea / constipation)	12 (11.8%)
Incomplete evacuation	7 (6.9%)
Subfertility	23 (22.5%)

The sigmoidoscopy procedure was completed satisfactorily in 95 (93.1%) of women. In 7 cases the procedure was incomplete; defined as a failure to achieve adequate views up to the splenic flexure. The reasons for failure were inability to tolerate the procedure (5 cases) and inadequate bowel prep (2 cases). The outcomes from flexible sigmoidoscopy are shown in Table III. A single case had luminal endometriosis suspected by the operator, however a biopsy of this area showed normal mucosa. There was a single case of suspected partial-thickness rectal wall involvement. The most common abnormal findings were non-specific evidence of rectovaginal nodules which included digital per rectum examination findings such as a palpable tender nodule, tightly angulated rectum and/or colon, and fixed sigmoid colon. There were no complications from the sigmoidoscopy procedures although 44.3% of patients reported moderate or significant discomfort. Histology from polyps and biopsy of suspicious areas revealed benign disease in all cases.

**Table III:** Sigmoidoscopy findings.

Pathology (N = 102)	n (%)
No abnormality	49 (48.0%)
Non-specific evidence of endometriosis	37 (36.3%)
Polyp	3 (2.9%)
Non-specific inflammation	2 (2.0%)
Ulceration	2 (2.0%)
Diverticulosis	1 (1.0%)
Haemorrhoids	1 (1.0%)
Incomplete assessment	7 (6.9%)

Following the sigmoidoscopy, all patients were counselled on the risk of bowel surgery including the possibility of stoma formation. The majority (98.0%) of patients underwent surgery follow-ing flexible sigmoidoscopy with 2 patients opting to continue with medical management only. In both cases this decision did not appear to be linked to the findings at sigmoidoscopy, where the investigation was reported as normal in both cases. The surgical findings and procedures includ-ing bowel surgery are shown in Table IV. The overall risk of requiring surgery directly to the bowel (bowel resection, disc resection and serosa repair) was 8% in this series. Of the 9 cases where rectovaginal DE was not confirmed at laparoscopy, all had prior imaging or diagnostic laparoscopy at other units suggestive of rectovaginal DE. In 2 of these cases, the prior sigmoidoscopy had shown non-specific findings for rectovaginal disease (tight angulation of the bowel) and in 7 cases the sigmoidoscopy had been completely normal. Of the 6 cases of bowel and disc resection, 5 had prior sigmoidoscopy with non-specific findings suggestive of endometriosis (tight angulation in every case) and 1 had a normal sigmoidoscopy).

**Table IV:** Surgical findings and procedure. In 9 cases DE was suspected pre-operatively but was not found at the time of surgery. “Other procedure” is defined as all procedures where the primary procedure did not involve rectovaginal nodule resection or hysterectomy. Rectal shave refers to the technique maintaining a dissection plane as superficially as possible on the rectum to avoid compromising the bowel integrity. Disc resection of the bowel involves removal of the endometriotic nodule infiltrating the bowel, followed by excision of a full thickness disc of tissue and primary closure of the defect.

Surgical Findings (N = 102)	n (%)
Rectovaginal DE confirmed	91 (89.2%)
Endometrioma only	3 (2.9%)
Superficial endometriosis only	3 (2.9%)
No evidence of endometriosis (scar tissue only)	3 (2.9%)
Declined surgery following sigmoidoscopy	2 (2%)
Primary Surgical Procedure (n=100)	
Laparoscopic excision of rectovaginal nodules only (i.e., no hysterectomy)	65 (65%)
Laparotomy and excision of rectovaginal nodules	1 (1%)
Laparoscopic hysterectomy (+/- BSO) and excision of rectovaginal endometriosis	19 (19%)
Laparotomy hysterectomy (+/-) and excision of rectovaginal endometriosis	7 (7%)
Other Procedure (ovarian cystectomy, treatment of superficial endometriosis, myomectomy)	8 (8%)
Bowel Procedures (n=92)	
Bowel Serosa injury and repair	2 (2.0%)
Bowel Resection	4 (4.1%)
Rectal Shave	84 (91.3%)
Disc resection	2 (2.0%)

## Discussion

The management of rectovaginal DE is complex and is best undertaken by a specialist multidisciplinary team (Ugwumadu et al., 2017; Keckstein et al., 2020). Surgery has traditionally been seen as the mainstay of treatment (Hoo et al., 2017) and despite some evidence showing good results from medical therapy alone (Wild et al., 2019), surgery will be indicated for many women. Surgery for rectovaginal DE aims to completely excise all visible endometriosis and is reported to have good long-term outcomes with significant improvements in symptoms and quality of life (Mallick, 2017; Byrne et al., 2018). Due to the complexity of surgery and the risk of significant morbidity associated with the potential for bowel resection and possible stoma formation, thorough pre-operative investigation and a comprehensive consent process is indicated. Pre-operative investigation should therefore aim to detail the extent and location of the disease, to aid surgical planning and patient counselling.

The pre-operative sigmoidoscopy is intended to detect the presence of luminal endometriosis or other evidence of bowel involvement such as narrowing or tight angulation to predict the need for bowel resection. For this reason, it has been the standard practice in our multidisciplinary endometriosis unit prior to embarking on surgery for rectovaginal endometriosis. Potential benefits include more confidence that the lesion does not involve the full thickness of the bowel wall when excising a rectovaginal nodule. This latter consideration may be especially useful to the surgeon when considering how to achieve complete resection of the nodule with the least morbidity possible. Less infiltrative disease is more amenable to rectal shaving or disc resection without the need for segmental bowel resection. These techniques have been shown to result in fewer complications such as rectovaginal fistulas, anastomotic leakage, delayed haemorrhage, and long-term bladder catheterisation when compared to segmental bowel resection (Donnez and Roman, 2017). Secondly, identifying the position of a full thickness lesion in relation to the dentate line is helpful pre-operatively as this would determine the likelihood of requiring a low or high anterior resection. A lower anterior resection has a significantly higher anastomotic leak rate (Vignali et al., 1997; Trencheva et al., 2013) along with a higher risk of bowel dysfunction and the development of low anterior resection syndrome (Wu et al., 2018). Therefore, knowledge of a full thickness lesion confirmed at sigmoidoscopy may have a role in aiding shared decision making and preoperative counselling. Finally, flexible sigmoidoscopy also has a useful role in excluding other pathology which may present in a similar manner to endometriosis, such as colonic malignancy or inflammatory bowel disease. Certainly, if rectal bleeding (haematochezia) is reported, endoscopy is usually indicated (Keckstein et al., 2020). In this study, haematochezia was reported by only 12.7% of patients. There were no incidental findings of malignancy or other serious pathology at sigmoidoscopy.

However, questions have been raised regarding the value of routine flexible sigmoidoscopy in this setting due to low rates of positive findings, patient discomfort and improving alternative imaging modalities. Although flexible sigmoidoscopy is generally a safe and well tolerated procedure, it invariably causes some discomfort and there is some evidence that the procedure is less well tolerated in a female population (Boltin and Niv, 2011). In this cohort, 44% patients reported moderate or severe discomfort. Finally, performing a sigmoidoscopy in every case could potentially lead to an unnecessary delay in surgical treatment for the patient.

This study did not find any confirmed cases of luminal bowel endometriosis out of 102 sigmoidoscopies performed in patients suspected or known to have rectovaginal DE based on prior investigations. This finding supports other studies which detail a very low incidence of luminal endometriosis in patients with rectovaginal DE of 1.7- 4.5% (Celentano et al., 2020; Lukovich et al., 2017; Milone et al., 2015). Furthermore, the ability of pre-operative sigmoidoscopy to accurately detect mucosal endometriosis may be limited. Recognised endoscopic findings of colorectal endometriosis include luminal angulation, luminal narrowing or extrinsic compression of the bowel lumen, polyps or masses, and mucosal changes such as erythema and granularity. However, only approximately 50% of suspected endometriotic lesions seen on colonoscopy will be confirmed as endometriosis (Kim et al., 2011) and it is accepted that a negative colonoscopy does not fully exclude the intramural presence of DE (Milone et al., 2015). To improve detection rates some authors suggest timing endoscopic assessment with menses when the endometriotic lesions are at their most prominent (Tanaka et al., 2021).

In many cases pre-operative sigmoidoscopy did reveal non-specific or “soft” signs of rectovaginal endometriosis such as a per rectal digitally palpable tender nodule, tight angulation or tethering of the bowel as noted by other authors (Lukovich et al., 2017). In general, in this series, the presence of “soft” signs did not help predict the likelihood of requiring a bowel resection during surgery (PPV = 11.8% and NPV = 96.9%) and therefore are likely to be of limited value. However, of note, the specific finding of tight angulation of the bowel was noted during sigmoidoscopy in all 4 cases which subsequently required a bowel resection. Tight angulation of the bowel may have some value in predicting the need for bowel resection although further research would be needed to determine if this is a consistent finding.

The patients in this study were routinely counselled prior to surgery on the risks of potential bowel surgery and the possibility of bowel resection. The nature of the advice given does not appear to have been significantly influenced by the findings at sigmoidoscopy. This is due to the fact that even with prior sigmoidoscopy alongside other investigations, it is very challenging to be sure of the depth and extent of bowel involvement from a given endometriotic lesion and it is usually only possible to know if and what type of bowel surgery is necessary at the time of the operation.

Due to the low incidence of luminal endometriosis in patients with known rectovaginal disease, performing sigmoidoscopy routinely is likely to be of questionable benefit and does not aid patient counselling in the vast majority of cases. However, there may still be a role for flexible sigmoidoscopy in selected cases. In this study 12.7% of patients presented with unexplained rectal bleeding and therefore would require investigation with flexible sigmoidoscopy (Keckstein et al., 2020). In total 8.8% of patients had a positive finding at endoscopy which was unrelated to endometriosis (polyps, ulceration, haemorrhoids).

A recent prospective study by Celentano et al. (2020) of 60 patients showed only 1 case of intraluminal endometriosis which was also suggested by MRI. The authors concluded they could not recommend the routine use of sigmoidoscopy for rectovaginal DE although they point out that the results are limited by small sample size. They suggest there could be value in performing flexible sigmoidoscopy in selected patients with rectal bleeding, change in bowel habit, family history of bowel cancer and inflammatory bowel disease (Celentano et al., 2020). Similarly, a prospective study of 174 women with DE calculated a sensitivity of 7%, specificity of 98%, positive predictive value of 85% and a negative predictive value of 58% for the detection of bowel endometriosis via endoscopy. The author noted that such a low sensitivity coupled with a low negative predictive value means bowel endometriosis cannot be reliably identified by endoscopy and a negative endoscopy does not exclude the need for bowel resection (Milone et al., 2015) and limits the value of endoscopy pre- operatively. Other authors suggest it’s use should be limited to specific rare cases of very large endometriotic nodules causing significant luminal bowel obstruction (Kim et al., 2011).

We would make the recommendation for selective flexible sigmoidoscopy, in two specific scenarios. Firstly, where serious colorectal pathology other than endometriosis is considered, such as proctocolitis or colorectal neoplasia. Secondly, where endoscopic assessment is deemed appropriate for determining the location of luminal endometriosis and subsequent resectional surgery planning. Outside of the above two scenarios, imaging in the form of ultrasound or MRI should be the primary investigation to aid diagnosis of rectovaginal DE and pre-operative planning. TVUS is often the first line investigation in patients with suspected rectovaginal DE as it is widely available and relatively inexpensive when compared to MRI. TVUS has similar diagnostic accuracy to MRI (Gerges et al., 2021) although MRI and is often preferred for complex cases with extensive adhesions (Chamié et al., 2011). MRI has been shown to be predictive of the need for bowel resection with lesions greater than 11mm size and bowel stenosis of 30% or greater suggesting an increased risk of resection (Scardapane et al., 2017). We are not aware of any published studies directly comparing the accuracy of sigmoidoscopy to imaging techniques such as MRI or TVUS for the diagnosis of bowel luminal endometriotic lesions or the ability to predict the need for bowel resection during surgery. Of note, endoscopy may also be combined with ultrasonography in the form of endoscopic ultrasound (EUS) which allows the bowel wall to be assessed for rectovaginal DE with high sensitivity (93.3%) and specificity (96.4%) reported (James et al., 2019) and offers a useful alternative to TVUS or pelvic MRI.

The main limitation of this study is the retrospective, observational design. The patient group were selected from a single tertiary referral unit and all procedures and operations were performed by the same team potentially limiting application of these findings to the wider population. Previous studies (Celentano et al., 2020; Lukovich et al., 2017; Milone et al., 2015), despite suffering from the same limitations, being either observational and retrospective (Lukovich et al., 2017) or with relatively small numbers of cases (Celentano et al., 2020; Milone et al., 2015) support the conclusions of our study that sigmoidoscopy is of limited value in this patient group.

## Conclusion

The findings from this study do not support the routine use of sigmoidoscopy in patients with rectovaginal DE due to the relatively low incidence of luminal disease, the relatively low sensitivity of sigmoidoscopy in detecting bowel endometriosis and the invasiveness of the procedure with associated patient discomfort and potential treatment delay. Furthermore, there would be significant cost savings from avoiding unnecessary procedures.

All current pre-operative investigations including sigmoidoscopy have a limited ability to determine the degree of bowel involvement in endometriotic nodules, meaning the final decision on the necessity and nature of bowel surgery can only be made at the time of surgery. Therefore, all patients with rectovaginal endometriosis should be counselled in a similar way prior to surgery and made aware that bowel surgery, including segmental resection in some cases, may be necessary to achieve excision of the endometriotic nodules. Following analysis of the data presented here, we no longer perform routine sigmoidoscopy for patients with rectovaginal DE prior to surgery and recommend selective use instead.

## References

[1] Abrão MS, Petraglia F, Falcone T (2015). Deep endometriosis infiltrating the recto-sigmoid: critical factors to consider before management.. Hum Reprod Update.

[2] Boltin D, Niv Y (2011). Curr Colorectal Cancer Rep. Is There a Place for Screening Flexible Sigmoidoscopy.

[3] Byrne D, Curnow T, Smith P (2018). Laparoscopic excision of deep rectovaginal endometriosis in BSGE endometriosis centres: a multicentre prospective cohort study.. BMJ Open.

[4] Celentano V, Di Donato N, Buccomino GE (2020). Prospective Evaluation of Outpatient Flexible Sigmoidoscopy in Patients With Deep Infiltrating Endometriosis.. Surg Laparosc Endosc Percutan Tech.

[5] Chamié LP, Blasbalg R, Pereira RMA (2011). Findings of pelvic endometriosis at transvaginal US, MR imaging, and laparoscopy.. Radiographics.

[6] Cornillie FJ, Oosterlynck D, Lauweryns JM (1990). Deeply infiltrating pelvic endometriosis: histology and clinical significance.. Fertil Steril.

[7] De Cicco C, Corona R, Schonman R (2011). Bowel resection for deep endometriosis: a systematic review.. BJOG.

[8] Donnez O, Roman H (2017). Fertil Steril. Choosing the right surgical technique for deep endometriosis: shaving, disc excision, or bowel resection.

[9] Gerges B, Li W, Leonardi M (2021). Meta-analysis and systematic review to determine the optimal imaging modality for the detection of uterosacral ligaments/torus uterinus, rectovaginal septum and vaginal deep endometriosis.. Hum Reprod Open.

[10] Giudice LC, Kao LC (2004). Endometriosis.. Lancet.

[11] Guerriero S, Ajossa S, Orozco R (2016). Accuracy of transvaginal ultrasound for diagnosis of deep endometriosis: systematic review and meta-analysis.. Ultrasound Obstet Gynecol.

[12] Guerriero S, Saba L, Pascual MA (2018). Transvaginal ultrasound vs magnetic resonance imaging for diagnosing deep infiltrating endometriosis: systematic review and metaanalysis.. Ultrasound Obstet Gynecol.

[13] Hoo WL, Hardcastle R, Louden K (2017). Management of endometriosis-related pelvic pain. Obstet Gynaecol.

[14] Keckstein J, Becker CM (2020). Recommendations for the surgical treatment of endometriosis. Part 2: deep endometriosis. Hum Reprod Open.

[15] James TW, Fan YC, Schiff LD (2019). Lower endoscopic ultrasound in preoperative evaluation of rectosigmoid endometriosis.. Endosc Int Open.

[16] Kim KJ, Jung SS, Yang SK (2011). Colonoscopic Findings and Histologic Diagnostic Yield of Colorectal Endometriosis.. J Clin Gastroenterol.

[17] Lukovich P, Csibi N, Brubel R (2017). [Prospective study to determine the diagnostic sensitivity of sigmoidoscopy in bowel endometriosis].. Orv Hetil.

[18] Mallick R (2017). The Diagnosis and Management of Severe Endometriosis.. MOJ Surg.

[19] Milone M, Mollo A, Musella M (2015). Role of colonoscopy in the diagnostic work-up of bowel endometriosis.. World J Gastroenterol.

[20] Nisolle M, Donnez J (1997). Peritoneal endometriosis, ovarian endometriosis, and adenomyotic nodules of the rectovaginal septum are three different entities.. Fertil Steril.

[21] Rolla E (2019). Endometriosis: advances and controversies in classification, pathogenesis, diagnosis, and treatment.. F1000Res.

[22] Scardapane A, Lorusso F, Francavilla M (2017). Magnetic Resonance Colonography May Predict the Need for Bowel Resection in Colorectal Endometriosis.. Biomed Res Int.

[23] Tanaka K, Sasaki A, Egashira H (2021). A Targeted Biopsy during Menstruation for the Definitive Diagnosis of Rectovaginal Endometriosis: A Report of Two Cases.. Intern Med.

[24] Trencheva K, Morrissey KP, Wells M (2013). Identifying Important Predictors for Anastomotic Leak After Colon and Rectal Resection.. Ann Surg.

[25] Ugwumadu L, Chakrabarti R, Williams-Brown E (2017). The role of the multidisciplinary team in the management of deep infiltrating endometriosis.. Gynecol Surg.

[26] Vignali A, Fazio VW, Lavery IC (1997). Factors associated with the occurrence of leaks in stapled rectal anastomoses: a review of 1,014 patients.. J Am Coll Surg.

[27] Wild M, Miskry T, Al-Kufaishi A (2019). Medical management of deeply infiltrating endometriosis - 7 year experience in a tertiary endometriosis centre in London.. Gynecol Surg.

[28] Wolthuis AM, Meuleman C, Tomassetti C (2014). Bowel endometriosis: colorectal surgeon’s perspective in a multidisciplinary surgical team.. World J Gastroenterol.

[29] Wu GJ, Jia WW, An Q (2018). Risk factors for low anterior resection syndrome.. Zhonghua Yi Xue Za Zhi.

